# Opposite effect of angiotensin receptor blockade on CXCL8 production and CXCR1/2 expression of angiotensin II-treated THP-1 monocytes

**DOI:** 10.3892/etm.2013.909

**Published:** 2013-01-21

**Authors:** KONSTANTINA VOGIATZI, STAVROS APOSTOLAKIS, ZAHARENIA VLATA, ELIAS KRABOVITIS, DEMETRIOS A. SPANDIDOS

**Affiliations:** 1 Laboratory of Clinical Virology, Faculty of Medicine University of Crete; Heraklion 71003, Crete, Greece; 2 Institute of Molecular Biology and Biotechnology, Department of Applied Bioschemistry and Immunology, Heraklion 71003, Crete, Greece

**Keywords:** Interleukin 8, THP-1 monocytes, angiotensin II, angiotensin receptor blockers

## Abstract

Interleukin-8 (IL-8) or CXCL8 is a potent chemotactic factor that is involved in atherogenesis. IL-8 mediates its pre-inflammatory effects through interaction with CXCR1 and CXCR2. In the present study, we investigated the effects of angiotensin II (Ang II) on IL-8 synthesis and CXCR1/CXCR2 expression of THP-1 monocytes. IL-8 was measured in the culture medium using ELISA. Expression of chemokine receptors CXCR1 and CXCR2 was evaluated by flow cytometry. Results demonstrated that the addition of Ang II increased IL-8 production in the THP-1 monocytes. The Ang II type 1 receptor blocker (ARB) losartan significantly blocked Ang II-induced IL-8 production. Notably, losartan blocked LPS-induced IL-8 production by THP-1 monocytes and produced a small but statistically significant reduction of baseline IL-8 production of naïve THP-1 cells. Losartan also produced a statistically significant increase of fluorescence intensity of naïve CXCR1- and CXCR2-positive THP-1 monocytes, probably as a negative feedback effect secondary to IL-8 downregulation. In conclusion, we demonstrated that Ang II increased IL-8 production by THP-1 monocytes. Losartan significantly suppressed the latter effect, suggesting an AT-1 mediated pathway. Moreover, losartan suppressed the IL-8 production of naïve THP-1 monocytes and LPS-treated THP-1 monocytes, suggesting a broader spectrum of pleiotropic effects. Extrapolating this *in vitro* observation to *in vivo* pathways, we propose Ang II-induced IL-8 production by monocytes as another pre-atherogenic potential of Ang II that can be effectively blocked by the AT1 receptor blockade.

## Introduction

Interleukin-8 (IL-8) or CXCL8 is a significant regulator of leukocyte trafficking and activation that results from the interaction with the cell surface receptors, CXCR1 and CXCR2 (1). In the field of vascular biology, monocytes/macrophages serve as the main source and primary target of IL-8. However, each cellular component of the vascular wall is able to produce IL-8 (1–3).

The renin-angiotensin system plays an important role in the initiation and progression of atherosclerosis (4). Angiotensin II (Ang II), the most active component of the renin-angiotensin system, has significant pre-inflammatory functions in the vascular wall, including the production of inflammatory cytokines and adhesion molecules (5,6).

The impact of Ang II on monocyte/macrophage-derived IL-8 has yet to be thoroughly investigated. We proposed that the pre-inflammatory properties of Ang II are not limited to vascular endothelium but are further expanded in circulating mononuclear cells. Thus, we hypothesized that Ang II significantly affects IL-8 production and/or significantly alters the CXCR1/CXCR2 phenotype of human monocytes/macrophages. To support our hypothesis, THP-1 monocytes were utilized to detect alterations of IL-8 production and CXCR1/CXCR2 surface expression in naïve cells and cells treated with Ang II. Pre-treatment with the angiotensin receptor blocker losartan was also applied to reveal the potential reversibility of AT-1 mediated effects.

## Materials and methods

### Cell cultures

THP-1 is a myelomonocytic cell line. THP-1 cells were cultured as previously described (7). In brief, RPMI-1640 medium supplemented with 10% decomplemented FBS and 2 mM glutamine, 25 mM HEPES, penicillin (50 U/ml) and streptomycin (50 U/ml) was used. Cells were cultured at a density of 500,000/ml, at 37°C, in a humidified 50 ml/l CO_2_ atmosphere. The chemokine receptor phenotype of the monocyte subpopulation was assessed by re-evaluating the mean fluorescence intensity (Geo Mean) and rate of chemokine receptor-positive cells in the monocyte gates of flow cytometer density plots.

Cells were treated with Ang II (Sigma-Aldrich, St. Louis, MO, USA) or lipopolysaccharide (LPS) in the presence or absence of Ang II type 1 receptor blocker (ARB) losartan or telmisartan. Three time points of 0, 24 and 48 h and concentrations of Ang II ranging from 0.2 to 20 *μ*M were initially evaluated. Losartan was evaluated in concentrations ranging from 10 to 1,000 *μ*M. Optimal results were obtained for 100 *μ*M of losartan. Bacterial LPS was used in a standard concentration of 10 ng/ml.

### Flow cytometry

The expression of chemokine receptors CXCR1 and CXCR2 was evaluated by flow cytometry using anti-CXCR1 fluorescein isothiocyanate-conjugated and anti-CXCR2 phycoerythrin-conjugated antibodies (BD Bioscience, Franklin Lakes, NJ, USA). Experiments were performed at least in triplicate and the mean fluorescence intensity ± standard deviation (SD) was reported. In all cases, the intra-assay coefficient of variation (CV) was <5% while the inter-assay CV was <10%.

### ELISA

Cells were seeded at a density of 500,000/ml. In the pre-set time points culture media were collected and centrifuged at 200 × g for 8 min to remove particles. The supernatants were frozen at −20°C until used for ELISA. The concentration of IL-8 was measured using an ELISA kit (R&D Systems, Minneapolis, MN, USA) according to the manufacturer’s instructions. Experiments were performed at least in triplicate and the mean concentrations (ng/ml) ± SD were reported.

### Statistical analysis

The paired sample t-test was applied to evaluate the differences between the means since this better eliminated bias attributed to the different baseline expressions of IL-8 among different experiments. P<0.05 was considered to indicate a statistically significant difference. Experiments were performed at least in triplicate (or as indicated by degrees of freedom at the reported results) and the mean concentrations ± SD or mean fluorescence intensity ± SD were reported.

## Results

The impact of the ARB losartan on IL-8 production and the CXCR1/CXCR2 phenotype of Ang II- and LPS-treated THP-1 monocytes is summarized in Table I.

### Interleukin 8 production

**Figure 1.**
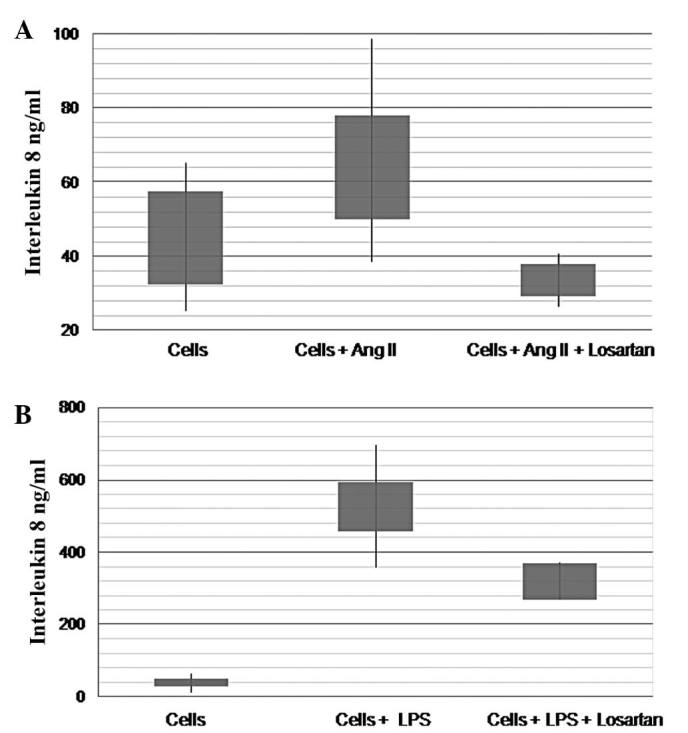
Box plots presenting range of values (boxes) and 95% confidence interval of means (lines) of interleukin 8 concentration in supernatant prior to and following treatment with (A) angiotensin II (Ang II) or Ang II plus losartan and (B) bacterial lipopolysaccharide (LPS) or LPS plus losartan. Cells represent the naïve state. Presented results refer to treatment with 10 ng/ml LPS, 10 *μ*M of Ang II and 100 *μ*M of losartan. All results were obtained 24 h post treatment.

Ang II produced a significant increase of IL-8 production by THP-1 monocytes. A maximum effect was achieved by 10 *μ*M of Ang II (45.2±12.5 vs. 68.8±18.9 ng/ml, df=3, t=−6.96, P=0.006) (Fig. 1B). LPS produced a similar but more pronounced effect (40.3±9.5 vs. 527±68.1 ng/ml, df=2, t=−14.2, P=0.005) (Fig. 1A). Similar results were obtained for the two substances in time points ranging from 12 to 48 h after treatment.

**Figure 2.**
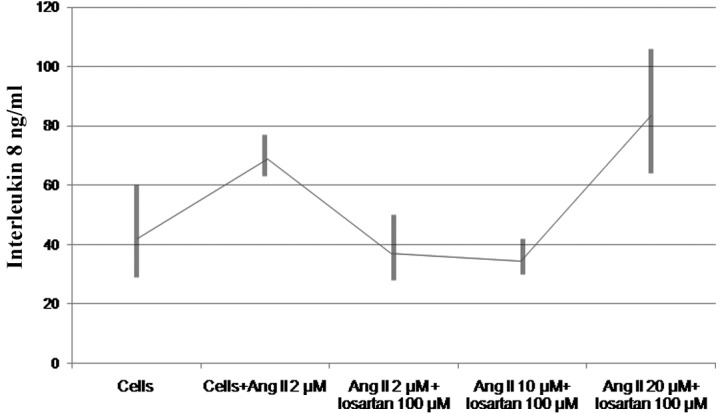
A graphic presentation of the impact of 100 *μ*M of losartan on the interleukin-8 production of THP-1 cells stimulated with various concentrations of angiotensin II (Ang II). Results refer to simultaneous incubation with 100 *μ*M of losartan plus various concentrations of Ang II. Cells represent the naïve state.

**Figure 3.**
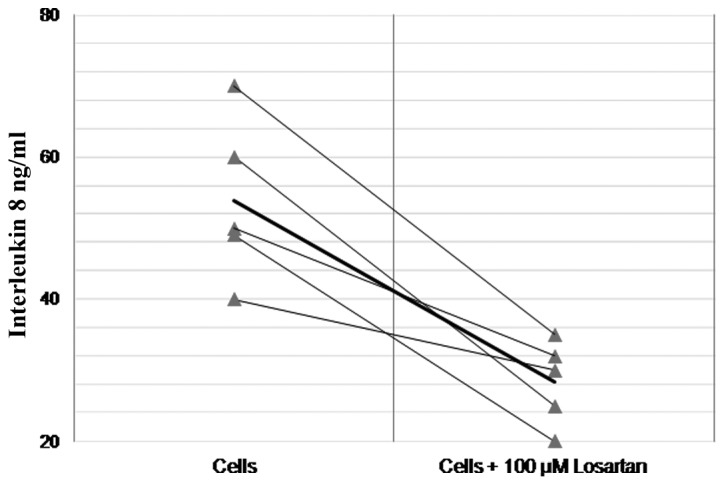
A graphic presentation of the impact of 100 *μ*M of losartan on the interleukin-8 production of non-LPS- or angiotensin II-stimulated THP-1 cells. Each thin line represents an individual experiment. The bold line shows the mean values.

Losartan significantly inhibited the effect of Ang II on the production of IL-8 by THP-1 monocytes (Fig. 1B). Losartan (100 *μ*M) successfully reversed the effect of 10 *μ*M or less of Ang II (76.7.8±12.6 vs. 36.0±4 ng/ml, df=2, t=8.2, P=0.015) (Fig. 2). The phenomenon was reproduced utilizing either a 2-h pretreatment with losartan or simultaneous incubation with Ang II and losartan (data not shown). Losartan significantly reduced the increase of IL-8 production induced by 10 ng/ml of LPS (527±68 vs. 320±20 ng/ml, df=2, t=7.3, P=0.018) (Fig. 1A). Additionally, losartan significantly reduced the baseline production of IL-8 in naïve (non-Ang II- or LPS-treated) THP-1 monocytes (58.3±28.4 vs. 28.4±5.9 ng/ml, df=4, t=5.1, P=0.006) (Fig. 3).

### CXCR1/CXCR2 phenotype

**Figure 4.**
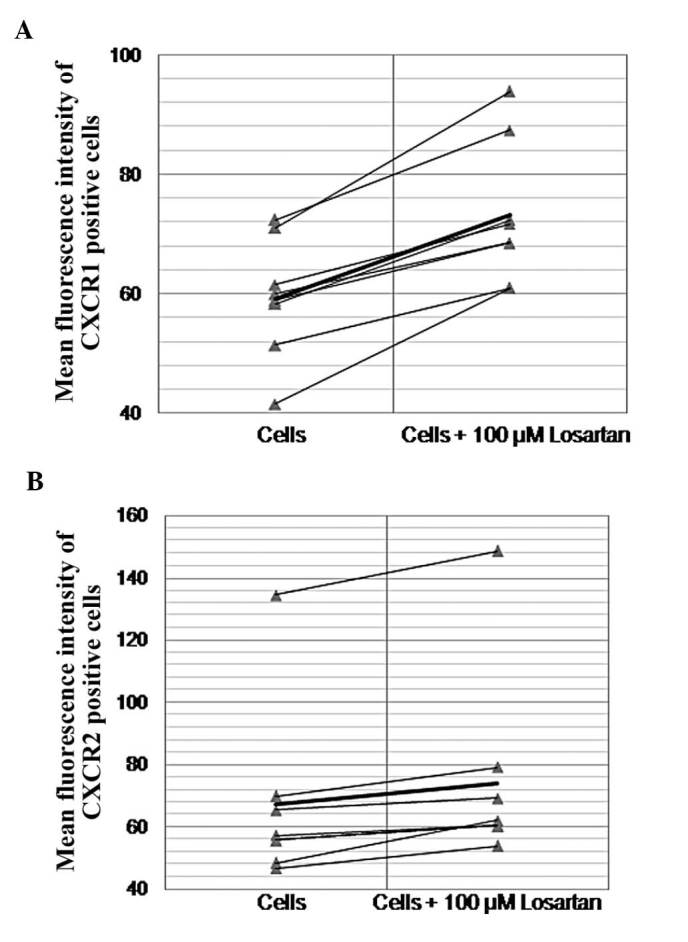
A graphic presentation of the impact of 100 *μ*M of (A) losartan CXCR1 and (B) CXCR2 fluorescence intensity (Geo Mean) of THP-1 cells. Each thin line represents an individual experiment. The bold line is the mean effect.

**Figure 5.**
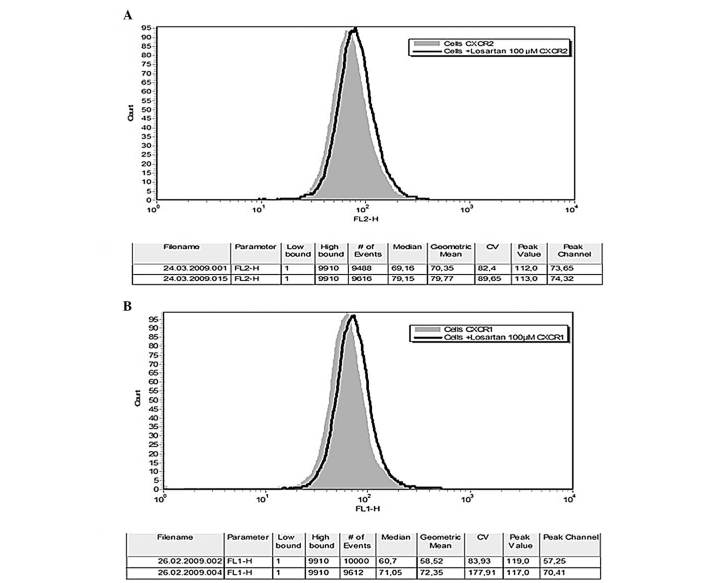
Representative histogram plots of (A) CXCR2 and (B) CXCR1 fluorescence intensity of THP-1 monocytes in the naïve state (grey area) and THP-1 monocytes treated with losartan (bold line).

Neither Ang II nor LPS affected the CXCR1/CXCR2 fluorescence intensity of THP-1 monocytes. Losartan significantly altered the CXCR1/CXCR2 phenotype of naïve or LPS or Ang II pre-treated THP-1 monocytes. Losartan (100 *μ*M) resulted in a small but constantly detected and statistically significant increase of the fluorescence intensity of CXCR1- and CXCR2-positive THP-1 cells (59.1±9.4 vs. 73.2±11, df=8, t=−8.4, P<0.0001 and 67.2±26.7 vs. 74±29, df=8 t=−4.19, P=0.003, respectively) (Figs. 4 and 5). In order to explore the possibility of a drug- instead of a class-effect, cells were also incubated with the ARB telmisartan before CXCR1 and CXCR2 fluorescence intensity was assessed. As with losartan, telmisartan increased the fluorescence intensity of CXCR1-positive cells (74±27.5 vs. 105±30.8, df=2, t=−9.6, P=0.01). However, no change was detected regarding the CXCR2 receptor (82.3±25.4 vs. 89.1±23, df=2, t=−2.1 P=0.17).

No effect was observed by the ACE captopril and lisinopril on IL-8 production by LPS- or Ang II-treated THP-1 cells.

## Discussion

There is sufficient amount of evidence in the scientific literature supporting the pre-inflammatory and pre-atherogenic properties of Ang II. In fact, most of the beneficial pleiotropic effects of the RAS blockade are attributed to the inhibition of Ang II-induced vascular damage (8,9). A considerable amount of evidence in this field has been derived from *in vitro* models that barely resemble actual biochemical pathways, but are able to identify an isolated cellular reaction to a particular stimulus at the biochemical and molecular level (10). In the present study, THP-1 monocytes were utilized for the study of Ang II effects on the activation of the IL8/CXCR1/2 pathway. The THP-1 cell line is a well-established model in the study of monocyte behavior since it shares many common characteristics with the normal human monocytes, including morphology, as well as the expression of plasma membrane receptors and cytokines (11). In the latter cell model, we demonstrated that Ang II significantly upregulated IL-8. ARB losartan attenuated this effect suggesting the existence of an AT-1-mediated pathway. We observed that losartan has the potential to attenuate LPS-induced IL-8 overexpression, a finding that supports the broader spectrum of losartan’s anti-inflammatory properties. In accordance with our observation, Chen *et al*(12) in similar settings, reported that Ang II elevated the levels of monocyte chemoattractant protein (MCP)-1, IL-8 and tumor necrosis factor-α and upregulated the CCR2 and CXCR2 mRNA expression of THP-1 monocytes. The authors further reported that pretreatment with losartan eliminated the effects mediated by Ang II. Similarly, Schmeisser *et al*(13) reported that the Ang II-induced upregulation of IL-8 and MCP-1 protein and RNA in monocytes was inhibited by the AT1R-blocker losartan. Ramiprilat was also found to suppress the Ang II-induced upregulation of IL-8 and MCP-1 in a dose-dependent manner. Thus, it appears that there is agreement on the effects of Ang II on IL-8 production by monocytic cells. However, our study further demonstrated that losartan treatment can reduce the baseline levels of IL-8 production and increase CXCR1/CXCR2 expression of cultured THP-1 cells. The latter observation opposes previously reported results supporting that Ang II upregulates IL-8 and its receptors and that losartan inhibits both effects (12,13). In this study, an opposite effect of losartan on IL-8 and CXCR1/2 receptors was observed. This is not the first time that such a phenomenon is observed. Reverse regulation of CXCR1 and/or CXCR2 in response to IL-8 alteration was previously reported in both *in vivo* and *in vitro* systems (14,15). Moreover, we previously observed and reported a similar effect of losartan on CX3CR1 expression of THP-1 monocytes, although the impact on the ligand was not assessed (7). The biochemical pathway leading to the losartan-induced upregulation of CXCR1/2 is obscure. Browning *et al*(16) provided direct evidence that autocrine IL-8 production occurs in monocytes stimulated with IL-8 and that this cell response is regulated at the receptor level. The authors assumed that the preferential usage of CXCR1 in autocrine IL-8 production occurs in certain types of cells, such as multinucleate cells. Samanta *et al*(17) reported on data suggesting that the IL-8 receptor expression is markedly regulated by IL-8. Since IL-8 regulates both its own and CXCR1/2 expression through CXCR1 activation, this opposite effect of losartan on IL-8 ligand and receptor expression could be attributed to the activation of auto-regulation pathways. Although no data are currently available to support this hypothesis, we observed a more pronounced losartan-induced increase in CXCR1 fluorescence intensity (CXCR1 is reported to be most actively involved in IL-8 auto-regulation). By contrast, this theory is opposed by the fact that a 10-fold increase of IL-8 induced by LPS did not affect the CXCR1/CXCR2 phenotype of THP-1 monocytes.

In conclusion, the *in vitro* model of this study demonstrated that Ang II increased IL-8 production by THP-1 monocytes through an AT-1-mediated pathway. ARB losartan attenuated both the Ang II- and LPS-induced overexpression of IL-8 and produced a small but statistically significant downregulation of baseline IL-8 production by THP-1 monocytes. Losartan also produced a small but statistically significant increase in the fluorescence intensity of CXCR1- and CXCR2-positive THP-1 cells. The biochemical basis of the latter observation deserves further investigation. Extrapolating this *in vitro* observation to *in vivo* pathways, we suggest Ang II-induced IL-8 production by monocytes as another pre-atherogenic potential of Ang II that can be effectively blocked by the AT1 receptor blockade.

## Figures and Tables

**Table I.** Effects of ARB losartan on the CXCR1/CXCR2 phenotype and Interleukin-8 (IL-8) production of LPS or Ang II treated THP-1 monocytes.

	LPS	Ang II	Losartan	Telmisartan	LPS+Losartan	Ang II+Losartan
CXCR1	↔	↔	↑	↑	↑	↑
CXCR2	↔	↔	↑	↔	↑	↑
IL 8	↑↑↑	↑↑	↓	o	↑↑	↔

↑↑↑/↓↓↓, >5-fold increase/decrease.

↑↑/↓↓, >2-fold increase/decrease.

↑/↓, any increase/decrease beyond the level of statistical significance.

↔, no statistically significant change.

○, not evaluated. Ang II, angiotensin II; LPS, bacterial lipopolysaccharide.
